# An Atraumatic Symphysiolysis with a Unilateral Injured Sacroiliac Joint in a Patient with Cushing's Disease: A Loss of Pelvic Stability Related to Ligamentous Insufficiency?

**DOI:** 10.1155/2016/9250938

**Published:** 2016-01-20

**Authors:** Andreas Höch, Philipp Pieroh, Faramarz Dehghani, Christoph Josten, Jörg Böhme

**Affiliations:** ^1^Department of Orthopedics, Trauma and Plastic Surgery, University of Leipzig, Liebigstrasse 20, 04103 Leipzig, Germany; ^2^Department of Anatomy and Cell Biology, Martin Luther University of Halle-Wittenberg, Grosse Steinstrasse 52, 06097 Halle (Saale), Germany

## Abstract

Glucocorticoids are well known for altering bone structure and elevating fracture risk. Nevertheless, there are very few reports on pelvic ring fractures, compared to other bones, especially with a predominantly ligamentous insufficiency, resulting in a rotationally unstable pelvic girdle. We report a 39-year-old premenopausal woman suffering from an atraumatic symphysiolysis and disruption of the left sacroiliac joint. She presented with external rotational pelvic instability and immobilization. Prior to the injury, she received high-dose glucocorticoids for a tentative diagnosis of rheumatoid arthritis over two months. This diagnosis was not confirmed. Other causes leading to the unstable pelvic girdle were excluded by several laboratory and radiological examinations. Elevated basal cortisol and adrenocorticotropic hormone levels were measured and subsequent corticotropin-releasing hormone stimulation, dexamethasone suppression test, and petrosal sinus sampling verified the diagnosis of adrenocorticotropic hormone-dependent Cushing's disease. The combination of adrenocorticotropic hormone-dependent Cushing's disease and the additional application of exogenous glucocorticoids is the most probable cause of a rare atraumatic rotational pelvic instability in a premenopausal patient. To the authors' knowledge, this case presents the first description of a rotationally unstable pelvic ring fracture involving a predominantly ligamentous insufficiency in the context of combined exogenous and endogenous glucocorticoid elevation.

## 1. Background

Besides their association with high-energy trauma, the majority (51%) of recent pelvic ring fractures are documented in patients older than 65 years after minor trauma, especially in female patients [1–5]. These fractures might occur in relation to an impaired bone stock and composition, for example, based on postmenopausal osteoporosis [5]. Other pathological conditions like rheumatoid arthritis with and without glucocorticoid (GC) therapy, malignancy, radiation, chemotherapy, drugs such as bisphosphonates, metabolic bone diseases, or pregnancy may contribute to pelvic ring fractures and a possible loss of stability [6–12].

Furthermore, these fractures may be present without adequate trauma or atraumatically and are often summarized as fragility fractures related to an impaired bone metabolism and composition [13]. Additionally, the manifold etiological factors emphasize the importance of functional fracture management [3, 14, 15]. Therefore, more detailed knowledge on the causes leading to atraumatic pelvic ring fractures is necessary. Apart from bone stability, the pelvic ligaments play a crucial role in preserving pelvic stability [16–21].

However, pathological conditions contributing to decreased ligament stability with an accompanied predominant atraumatic ligamentous fracture pattern are still scarce [22, 23]. Here, we report on a 39-year-old premenopausal female patient suffering from a spontaneous atraumatic symphysiolysis, an anterior lesion of the left sacroiliac joint, and a possibly related transiliac instability caused by an ilium fracture. After the exclusion of other causes leading to this injury the GC excess after two months of GC therapy and previously undiagnosed Cushing's disease (CD) seem to be the most plausible reasons contributing to this predominantly ligamentous insufficiency presenting as a rotationally unstable pelvic ring fracture.

## 2. Case Presentation

A 39-year-old woman was admitted to our department from a rheumatology clinic presenting with symphysiolysis and injured left sacroiliac joint. The patient complained about sudden incipient pain in the left hip while walking without a history of trauma. Previously, she was treated with 100 mg prednisolone for the tentative diagnosis of rheumatoid arthritis. The dose was reduced about 10 mg every fifth day and discontinued due to her increased pain, two months after starting the GC therapy.

At the time of admission to the rheumatologic department, the patient's height was 158 cm, she weighed 70 kg (body mass index [BMI] 28 kg/m^2^), and she had blood pressure of 110/80 mmHg. She presented with moon facies, abdominal obesity, no edema, no struma, atrophy of the interossei muscles of her hands, positive Gaenslen signs, and compression pain in both wrists, the metacarpophalangeal joints (MCP), distal thumb joint, and the forefeet. Moreover, her thumb saddle joints, the wrists, the right thumb MCP joint, and her left MCP II were swollen. The patient bore one child fifteen years before her admission to the hospital and had no menstruation disorders or other gynecological diseases.

**Figure 1:**
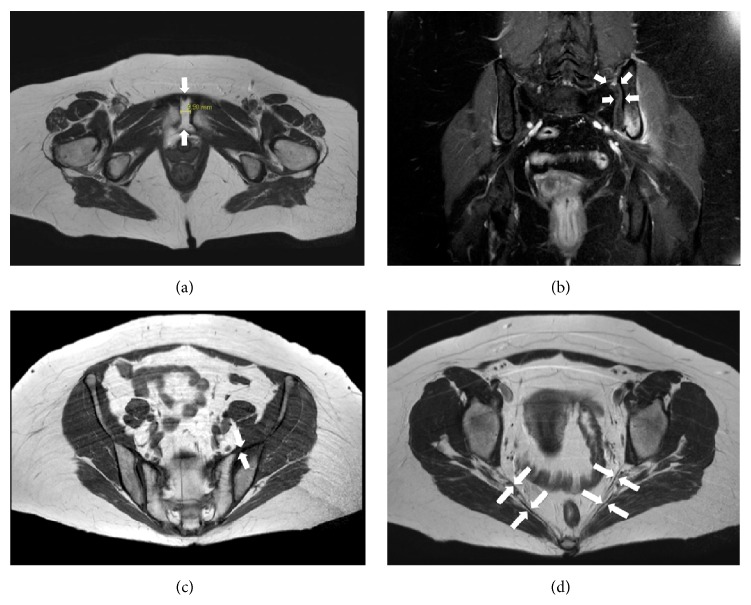
MRI of the pelvis. (a) Axial MRI presenting extensive widening of the symphysis about 9.9 mm (arrows). (b) Coronary MRI also showed widening of the left sacroiliac joint (arrows) and an increased signal from the left ilium. (c) Axial MRI revealed ruptured left anterior sacroiliac ligaments and intact posterior sacroiliac ligaments (arrows). (d) Axial MRI showed intact sacrotuberal and sacrospinal ligaments on both sides (arrows).

Laboratory examination revealed enhanced levels of white blood cells (WBC, 13.9 Gpt/L), C-reactive protein (CRP, 48.3 mg/L), bone alkaline phosphatase (ALP, 43.9 U/L), and basal cortisol (819 nmol/L) and normal levels of calcium, phosphorus, 1,25-OH vitamin D, thyroid-stimulating hormone (TSH), thyroxine (T_4_), parathyroid hormone (PTH), and uric acid (Table 1). Several tested autoantibodies were negative and all other investigated blood cell counts showed values within their specific references. For further investigations, magnetic resonance imaging (MRI) of the pelvis and the head, a bone scintigram, and a dual-energy X-ray absorptiometry (DXA) were performed. In the MRI of the head, no tumor and adenoma and especially no hypophyseal abnormalities were detected. The T1-weighted MRI of the pelvis showed widening of the left sacroiliac joint, an assumed fracture line of the left ilium running to the sciatic notch, and extensive widening of the symphysis to about 9.9 mm (Figures 1(a) and 1(b)). Additionally, the MRI revealed a disruption of the anterior sacroiliac ligaments on the left side and no affection of the posterior sacroiliac ligaments, the sacrotuberal and sacrospinal ligaments (Figures 1(c) and 1(d)).

**Figure 2:**
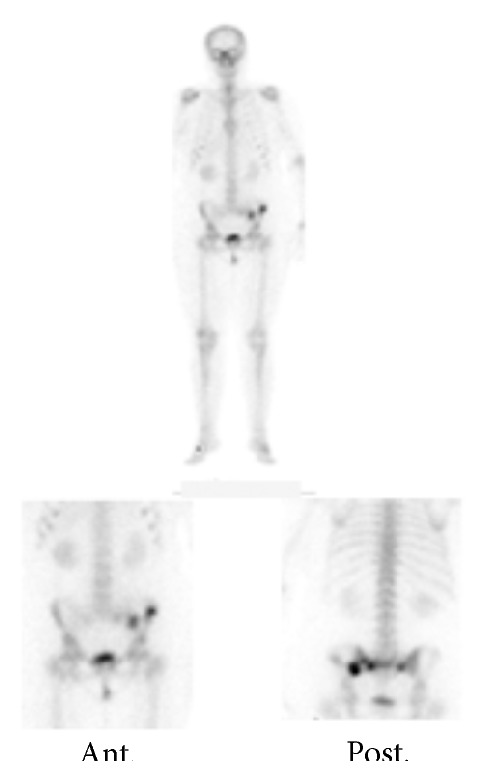
Bone scintigram of the pelvis revealed an increased uptake in the left ilium and in both sacroiliac joints.

**Figure 3:**
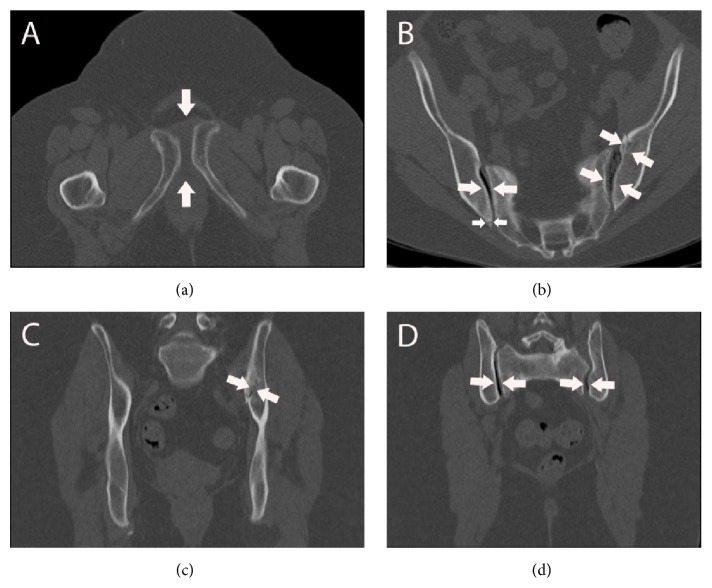
The CT-scan of the pelvic girdle confirms the observations of MR-image. (a) Axial CT-scan presented widening of the symphysis (arrows). (b) Axial CT-scan offered vacuum phenomenon in both sacroiliac joints, small tearing out in the left sacroiliac joint, and small dorsal fracture fragment in the right dorsal ilium (arrows). (c) Coronary CT-scan revealed the fracture line in the left ilium (arrows). (d) Coronary CT-scan presented vacuum phenomena in both sacroiliac joints (arrows).

The bone scintigram demonstrated an increased uptake in the left ilium and in both sacroiliac joints (Figure 2). The following computed tomography (CT) showed widening of the symphysis by about 12 mm and of the left sacroiliac joint by about 9 mm (Figures 3(a) and 3(b)). The left os pubis was shifted cranially by about 9 mm. Furthermore, a small tear in the left sacroiliac joint and vacuum phenomena in both sacroiliac joints were detected (Figures 3(b) and 3(d)). Furthermore, a small fracture fragment was detected at the right ilium (Figure 3(b)). In addition, to the fracture line of the left ilium observed in MRI (Figure 3(c)), fracture lines of the sacrum were found.

In the DXA *T*-scores of −2 for the left hip and −1.6 for the lumbar spine were measured, indicating osteopenia (Table 1). Based on these data, we determined the instability of the pelvis as an atraumatic rotational unstable pelvic ring fracture with a predominantly ligamentous insufficiency and a transiliac instability. Physical examination, laboratory tests, and imaging failed to establish a diagnosis of rheumatoid arthritis. Nevertheless, the surgical treatment of the unstable pelvis was recommended based on the patient's progressive pain and immobilization.

At the time of admission to our department, the patient presented with pelvic instability to external rotation and immobilization due to her progressive pain, predominantly localized at the sacrum and in the left sacroiliac joint. Additionally, she reported tenderness in the groin after palpation, predominantly on the left side. Taking into account the previously performed CT-scan and MRI, further X-rays were renounced, to reduce the radiation to our patient. Previously, X-rays of the left hip (a.p. and hip axial [Lauenstein I]) and an a.p. X-ray of the pelvis were done in the rheumatology clinic. However, the a.p. of the pelvis was performed with a protector for the gonads and thus the symphyseal diastasis could not be determined in an appropriate manner. Furthermore, single-leg stance X-rays were not possible, due to the patient's pain. The patient was treated with an unlocked plate at the symphysis and a triangular osteosynthesis of the posterior arch. Due to the untypical fracture pattern and related instability without a history of trauma, the authors classified the presented injury pattern as fragility fracture of the pelvis (FFP) type IIIb according to the recent published classification system of Rommens and Hofmann [13]. Moreover, several examinations were performed to exclude malignancy or other causes contributing to pelvic instability in an atraumatic manner. The thorax-abdominal CT, aspiration of the left sacroiliac joint, and intraoperative biopsies revealed no malignancy or inflammatory process. Gynecological examination yielded no indication for pregnancy or malignancy.

The analyzed blood samples showed no evidence of change in bone metabolism, malignancy, inflammatory processes, or endocrinological changes. However, laboratory results showed an increase of adrenocorticotropic hormone (ACTH, 12.58 pmol/L; Table 1). The remaining laboratory parameters revealed values within their specific reference values. To clarify the cause of the high cortisol and ACTH levels, a dexamethasone suppression test was performed and yielded no distinct suppression. Therefore, further diagnostic procedures were performed in our endocrinological department, including the repetition of the dexamethasone test. Here, the patient displayed pathological midnight cortisol and ACTH levels (cortisol 493.8 nmol/L, ACTH 14.41 pmol/L). Moreover, upon corticotropin-releasing hormone (CRH) stimulation, pathological increases of cortisol and ACTH were determined (Table 2). However, the dexamethasone suppression test presented missing suppression after 8 mg of dexamethasone (cortisol 627.8 nmol/L, ACTH 14.72 pmol/L). Petrosal sinus sampling was performed to confirm the diagnosis of a central CD and revealed a central-to-peripheral ACTH gradient (cavernous/peripheral, Table 2). Hence, the diagnosis of an ACTH-dependent CD was confirmed.

## 3. Case Discussion

We report on a 39-year-old premenopausal woman who suffered from symphysiolysis and a disruption of the left sacroiliac joint without a history of trauma. Other reasons contributing to an unstable pelvic girdle were excluded [6–12]. Hence, the most plausible reason for the presented injury pattern might be the combination of the endogenously increased GCs related to CD and the further enhancement of GC by the initial high-dose prednisolone therapy. The effects of GC and related fractures are generally associated with an impaired bone stock and/or composition. However, the present case impresses with a predominantly ligamentous insufficiency which is rare after GC elevation and also for atraumatic and fragility fractures [13, 15, 24].

### 3.1. Exogenous and Endogenous GC Effects on the Musculoskeletal System

GCs are well known to enhance the fracture risk by decreasing the number of osteoblasts and osteocytes, reducing osteoblastic function and production of extracellular matrix, and thus may lead to the loss of pelvic stability [22, 25–31]. Hence, bone mass decreases and fracture risk increases [26, 32]. The GC-related fracture risk depends essentially on initial dose, duration of GC therapy, and gonadal state. Thus, postmenopausal women displayed a significantly higher risk of experiencing a GC-induced fracture when compared to premenopausal women [32]. Within the first 12 months of GC therapy the highest bone loss is reported and in premenopausal women a bone loss of approximately 10% at iliac crest was detected six months after starting a high-dose GC therapy [29, 32]. Related to this bone loss, bone mineral density (BMD) is also decreased [28]. Nevertheless, the fractures related to GC more frequently occur in the time frame between 12 and 18 months after starting the therapy [27, 29, 32]. However, the effect of GC inducing a ligamentous insufficiency was not reported. Beyond the high-dose GC therapy, our patient presented a further disease increasing GCs endogenously, namely, a central CD. CD leads to similar changes of the musculoskeletal system compared to exogenous GC like muscle weakness, decreased bone load, and degradation of collagen associated with loss of BMD during CD [23, 33, 34]. Although osteoporosis is a common complication of CD [23] the BMD in premenopausal women is often not decreased in comparison to postmenopausal women [23, 35, 36]. Furthermore, ligamentous tissue is sensitive to cortisol and ACTH. The injection of cortisol in the posterior cruciate ligament led to decreased strength values without changing the ligament's stiffness [34]. Moreover, ACTH reduced strength values and fiber thickness of ligamentous tissue [37]. However, to clearly determine a solely CD-related fracture is very difficult as the majority of CD patients are treated with GC following the primary treatment to attenuate changes of other hormonal axes [38]. Additionally, CD patients often experience fractures two years prior to the diagnosis. Thus, a differentiation between endogenously (CD) or exogenously (GC therapy) induced fractures is difficult to make. Moreover, the BMD seems to be unreliable in detecting a GC-related osteoporosis by the occurrence of vertebral fractures in GC treated patients independent of their BMD [26]. A further issue is presented in the association of GC-related fractures in the time frame occurring after starting GC therapy. Although the majority of fractures occurred between 12 and 18 months, the risk rate in the first three months of 2 out of 100 persons per year is very high [27].

Considering these data, our case revealed some discrepancies in the presented injury pattern as when compared with a typically GC-related fracture. Our patient received the GC therapy only for two months and is premenopausal. However, the CD might have been present previously and GC therapy could have exacerbated the GC-related fracture risk. Nevertheless, as described above, the majority of GC effects are associated with an impairment of bone stock and composition, but this case involves an injury that is predominantly based on a ligamentous insufficiency, especially at the anterior arch of the pelvic girdle. Previous reports on GC-induced pelvic ring fractures are still scarce or related to a long GC therapy treating rheumatoid arthritis in postmenopausal women or male CD patients [22, 23]. But so far, in these reports, ligament insufficiency has not been described.

### 3.2. Injury Description

There are few reports in the literature with comparable injury patterns and no history of trauma. These cases are documented in patients with rheumatoid arthritis and a long history of GC therapy or postmortem in CD patients [22, 23, 31].

The injury pattern of our patient is characterized by an atraumatic symphyseal diastasis with a widened left sacroiliac joint, a rupture of the left anterior sacroiliac ligaments, and a transiliac instability manifested as ventral ilium fracture. Indeed, the application of currently used classification systems for pelvic ring fractures, based on the force leading to the fracture and the corresponding trauma, is restricted by the absence of an adequate trauma and classical signs for these fractures [16, 39]. Thus, the injury pattern of our patient is most likely classified as fatigue or fragility. Based on the rarity of atraumatic pelvic ring fractures, a specific classification system for these fractures is still lacking [40].

However, Rommens and Hofmann published a classification system for fragility fractures of the pelvis which can be used. According to this system, the fracture would be classified as fragility fracture of the pelvis type IIIb [13]. Furthermore, there are some reports comparable regarding an anterior instability impairing the posterior arch [41–44]. Causes for anterior instability are related to septic arthritis of the symphysis, wedge resection of the symphysis treating osteomyelitis pubis, or a nonunion of pubic arch fractures. Here, the compromised stability of the anterior arch might lead to the observed sacral or ilium fractures without an additional trauma. Indeed, our patient did not suffer from such circumstances, but the instability might be caused by the symphyseal diastasis which was also reported after long-term therapy with GC treating rheumatoid arthritis [22]. As reported by Dommisse the strength of the anterior pelvic ring stability depends on the stability of the symphyseal complex, especially on the anterior interpubic ligament [45]. An impairment of this complex might lead to posterior arch affection by the reduced resistance of the anterior sacroiliac ligaments withstanding the increasing forces contributing to the rupture of these ligaments. Considering this hypothesis revealed a possible explanation for the injury pattern in our case and also for the above mentioned cases [41–44]. Hence, the mobility of the sacroiliac joint is enhanced and may lead to a transiliac instability based on sacroiliac hypermobility [22]. Thus, the stabilization of the anterior pelvic ring might be recommended [45, 46]. Indeed, the instability seems to be smaller compared to APC and “open book” fractures because classical signs for instability like the rupture of the sacrospinal, sacrotuberal ligaments and a symphyseal diastasis greater than 2.5 cm are missing [16]. However, the sacrospinal and sacrotuberal ligament seem to have a minor role contributing to pelvic stiffness and vertical or horizontal displacement [17, 47]. Moreover, their rupture might not be ubiquitous as observed in recent finite element and MRI studies [19, 20, 48]. Hence, the rupture of the sacrospinal and/or sacrotuberal ligaments may not be a pivotal criterion describing the presented instability as well as the symphyseal displacement. Regarding the symphyseal diastasis a recent study revealed a reduced reliability of this parameter investigating APC type I fractures using stress examinations [49]. This study presented in 30% of patients a decisive underestimation of the instability comparing plain radiographs and findings of stress examinations leading to an upgrade of the instability after stress examinations. Moreover, biomechanical tests revealed ligamentous injuries possible between 1 and 4.5 cm [50]. Hence, this parameter might not be as reliable as MRI to investigate the ligamentous injury and related instability [48]. Nevertheless, the presented injury seems to be related to a ligamentous insufficiency. This assumption is supported by finite element studies demonstrating a predominant fracture region presented in high tensile stress zones in the ilium near to the sciatic notch, comparable to the fracture observed here [18]. Indeed, the fracture might lead to a presumptive crescent fracture also reported in the aftermath of anterior instabilities [44]. However, a crescent fracture presents with a dorsal facture line starting from the sacroiliac joint running to the iliac crest but a diastasis of the symphysis was not described [51]. Moreover, in the presented case, a bony affection of the sacroiliac joint is not determined.

Beside the traumatic, infectious, inflammatory, or surgical impairment of pelvic ligament stability or anterior arch stability, this situation might occur in pregnancy as a rare complication after delivery [52, 53]. Related to the hormonal changes during pregnancy, the symphysis is widened up to 10 mm [54–56]. There are some reports describing immobilization, low back pain, and pelvic tenderness occurring during the first 24 hours after delivery with a widened symphysis [56]. These reports did not describe an affection of sacroiliac joint. However, MRIs of these patients were not presented and thus a possible injury could not be excluded. In addition, a few days to three months after delivery, the symphysis returns to a normal size under bed rest and reduced weight-bearing which might be related to a compensation of hormonal axes like a decrease of relaxin levels [57]. Moreover, the process of delivery may also represent a kind of trauma. In contrast to these circumstances a permanent ACTH elevation did not return spontaneously and might have led to prolonged fracture healing, impairing stability and mobility [58]. Nevertheless, our patient was not pregnant and her delivery was 15 years ago.

## 4. Conclusion

Considering the data, the patient suffered from a symphyseal diastasis leading to widening of the left sacroiliac joint with a rupture of the left anterior sacroiliac ligaments and by the hypermobility of the sacroiliac joint to a consecutively transiliac instability manifested as ventral ilium fracture. Comparable cases were previously reported caused by infectious or inflammatory changes to the symphysis and the adjacent pubic bone, after wedge resection of the symphysis or long-term GC therapy treating rheumatoid arthritis [41–44].

Due to the absence of a fracture line running to the sacroiliac joint, we could not determine a crescent fracture [51]. It seems to be more likely that ligamentous insufficiency contributed to the described ilium fracture as supported by finite element analysis [18]. Indeed, the instability appears not as serious as reported for APC type II/III or “open book” fractures by missing classical signs of instability like ruptured sacrospinal and/or sacrotuberal ligaments and less symphyseal displacement [16, 39]. However, recent trials revealed the nonubiquity of ruptured sacrospinal/sacrotuberal ligaments and their minor role contributing to pelvic stiffness and vertical and horizontal displacement as well as the reduced reliability of measured symphyseal displacement on plain radiographs [17, 19, 20, 47–49]. Thus, the related instability cannot be deduced from these signs, especially in this case of an atraumatic etiology. The most plausible cause for the presented rare atraumatic rotational pelvic instability in a premenopausal patient seems to be the combination of an undetected central CD with elevated ACTH levels and the parallel application of exogenous GCs leading to ligament instability and loss of bone mass. Although we are not able to clearly determine if CD or the GC therapy, related to their simultaneous elevation of GCs, led to this injury, the duration of GC therapy in our patient seems to be too short to induce a fracture based on GC therapy. In addition, most publications determined the increased fracture risk after GC therapy to changes of bone stock and composition whereas our patient suffered from osteopenia and a predominantly ligamentous insufficiency [38]. Moreover, we are not able to clearly determine if ACTH affects the pelvic ligaments. However, previous studies determined a decrease in ligament strength related to ACTH treatment and present thereby a plausible explanation for the observed ligament-induced loss of pelvic stability [34, 37]. Indeed, reports with comparable cases and similar etiology are still scarce and describe such injuries after long-term GC therapy treating an inflammatory disease, namely, rheumatoid arthritis, or as postmortem findings in male CD patients [22, 23].

To summarize, this case presented the loss of the pelvic ligaments' stability as a possible cause of pelvic fracture, underlining their essential role in preserving pelvic stability and the not entirely known effects of medication and hormones on this kind of tissue. Additionally, the presented case shows a new manifestation of GC- and ACTH-induced fractures lesions and offers new insights into etiological factors contributing to pelvic fractures. Furthermore, the case supports findings of anterior instability leading to an impairment of the posterior arch [41–44].

## Figures and Tables

**Table 1:** Summary of blood examination and DXA.

WBC	17.80 Gpt/L	ALB	63.20 g/L

Hb	8.70 mmol/L	CR	73 *μ*mol/L

ESR	29 mm	Na	143.30 mmol/L

CRP	48.20 mg/L	K	3.92 mmol/L

ALP	43.90 U/L	P	1.21 mmol/L

ALT	0.49 *μ*kat/L	Ca	2.35 mmol/L

ASAT	0.46 *μ*kat/L	Cortisol	819 nmol/L

TSH	0.45 mU/L	ACTH	12.58 pmol/L
		1,25-OH vitamin D	27.30 ng/mL

*T*-score			

Left hip	−2.0		

Lumbar spine	−1.6		

**Table:** (a) CRH stimulation test

Time (min)	−15	0	15	30	45	60

Cortisol (nmol/L)	411.2	514.5	857	869.9	811.1	730.3

ACTH (pmol/L)	9.83	11.59	28.22	24.25	20.56	16.98

**Table:** (b) Sinus petrosus sampling

Right sinus petrosus					

Time (min)	−10	0	3	5	10

ACTH (pmol/L)	477.2	492.9	3024	2247	1815

Left sinus petrosus					

Time (min)	−10	0	3	5	10

ACTH (pmol/L)	86.04	46.82	546.2	486.7	232.5

Peripheral venous					

Time (min)	−10	0	3	5	10
ACTH (pmol/L)	11.51	12.88	17.18	20.74	23.99
